# The polysialic acid mimetics 5-nonyloxytryptamine and vinorelbine facilitate nervous system repair

**DOI:** 10.1038/srep26927

**Published:** 2016-06-21

**Authors:** Vedangana Saini, David Lutz, Hardeep Kataria, Gurcharan Kaur, Melitta Schachner, Gabriele Loers

**Affiliations:** 1 Department of Biotechnology, Guru Nanak Dev University, GT Road, 143005 Amritsar, India; 2 Center for Molecular Neurobiology, University Hospital Hamburg-Eppendorf, Martinistrasse 52, D-20246 Hamburg, Germany; 3 Keck Center for Collaborative Neurosciences, Rutgers University, Piscataway, NJ 08854, USA; 4 Center for Neuroscience, Shantou University Medical College, Shantou, Guangdong 515041, People’s Republic of China

## Abstract

Polysialic acid (PSA) is a large negatively charged glycan mainly attached to the neural cell adhesion molecule (NCAM). Several studies have shown that it is important for correct formation of brain circuitries during development and for synaptic plasticity, learning and memory in the adult. PSA also plays a major role in nervous system regeneration following injury. As a next step for clinical translation of PSA based therapeutics, we have previously identified the small organic compounds 5-nonyloxytryptamine and vinorelbine as PSA mimetics. Activity of 5-nonyloxytryptamine and vinorelbine had been confirmed in assays with neural cells from the central and peripheral nervous system *in vitro* and shown to be independent of their function as serotonin receptor 5-HT_1B/1D_ agonist or cytostatic drug, respectively. As we show here in an *in vivo* paradigm for spinal cord injury in mice, 5-nonyloxytryptamine and vinorelbine enhance regain of motor functions, axonal regrowth, motor neuron survival and remyelination. These data indicate that 5-nonyloxytryptamine and vinorelbine may be re-tasked from their current usage as a 5-HT_1B/1D_ agonist or cytostatic drug to act as mimetics for PSA to stimulate regeneration after injury in the mammalian nervous system.

Polysialic acid (PSA) is a homopolymer of 8 to 200 alpha-2,8-glycosidically linked sialic acid residues attached predominantly to complex N-glycans in the fifth immunoglobulin-like domain of the neural cell adhesion molecule (NCAM)^1^. In mammalian cells, two polysialyltransferases, ST8SiaII and ST8SiaIV, specifically add PSA chains to the terminal sialic acid residues of N- or O-linked glycans. PSA chains comprising more than 90 sialic acid residues lead to polyanions (negatively charged carboxylate groups) with high water binding capacity and the large hydration shell formed increases the hydrodynamic volume of its carrier molecules^2^. In the developing and adult nervous system of higher vertebrates, PSA is expressed by migrating cells, like olfactory interneuron precursors, by dynamically extending processes of cells such as neurons or Schwann cells, in regions of synaptic plasticity, and by stem cells, e.g. in the subventricular zone^3^,^4^,^5^. PSA promotes cell motility and axonal pathfinding and targeting, is implicated in learning and memory as well as synaptic plasticity, and it mediates the interaction of NCAM with other molecules, such as heparin sulfate proteoglycans^6^, brain derived neurotrophic factor^7^, α-amino-3-hydroxy-5-methyl-4-isoxazolepropionic acid receptors^8^, N-methyl-D-aspartate receptors^9^, histone H1^10^ and myristoylated alanine-rich C kinase substrate^11^.

These functions of PSA are of interest for treatment of nervous system injuries and disorders. Overexpression of PSA by astrocytes improves axonal regrowth across spinal cord injuries^12^. Viral-induced expression of PSA enhances regeneration after spinal cord injury^13^, promotes sensory neuron integration into the injured spinal cord^14^, and increases Purkinje cell dendrite formation following injury^15^. Application of transplanted Schwann cells overexpressing PSA or application of the PSA mimicking peptides or the PSA mimicking small organic compound tegaserod augment repair in both spinal cord^16^,^17^,^18^ and peripheral nerve injuries^19^,^20^,^21^. Application of PSA-NCAM-positive neural precursor cells in a rat stroke model improved survival, differentiation, and integration of these cells and suppressed adverse glial activation and angiogenesis^22^. However, a dosage- and time-constrained approach to administration of PSA must be considered as continuously expressed PSA slows down the rate of myelination *in vivo*^12^,^23^, and high PSA expression is correlated with invasiveness and malignancy of cancers^24^.

Purification difficulties and degradation of PSA by mammalian sialidases *in vivo* hamper the administration of PSA or its bacterial analog colominic acid, but progress has been achieved with mimetics of PSA. Peptide mimetics and small compound mimetics of PSA improved functional recovery following peripheral nerve and spinal cord injuries^17^,^18^,^20^,^21^,^25^. These peptides and small compound mimetics of PSA offer advantages for development and approval of therapies applicable in humans. Purified synthetic compounds are non-xenogeneic as compared to proteins or molecules purified from animals and bacteria or recombinantly generated in *E. coli*, insect or mammalian cells. To build upon the previous advances of identifying PSA mimetics with translational potential, we have screened a library of small organic molecules to identify novel PSA mimetics. This screen identified the compound 5-nonyloxytryptamine oxalate (5-NOT) and the FDA approved third generation vinca alkaloid vinorelbine as PSA mimetics, and both compounds stimulated neuronal functions beneficially *in vitro*^26^,^27^, thus encouraging the evaluation of their capacities *in vivo*.

In the current study, we compared the effects of 5-NOT and vinorelbine with colominic acid on the functional, histological and ultrastructural outcome after spinal cord compression injury in mice. 5-NOT, vinorelbine and colominic acid treatment led to enhanced recovery of mice following spinal cord injury. The extent of regeneration was comparable in 5-NOT and colominic acid treated mice and less pronounced in vinorelbine treated animals. 5-NOT, vinorelbine and colominic acid promoted axonal regrowth, motor neuron survival, density of glutaminergic synapses on motor neurons. Both compounds and colominic acid also facilitated remyelination of axons and cholinergic innervation caudal to the lesion site as well as enhanced axonal diameters and thickness of myelin sheaths. These studies are promising in that especially 5-NOT may be re-tasked to nervous system therapies, possibly facing fewer difficulties in regulatory approval and clinical translation.

## Results

### Application of 5-NOT and colominic acid improves recovery after spinal cord injury

**Figure 1:**
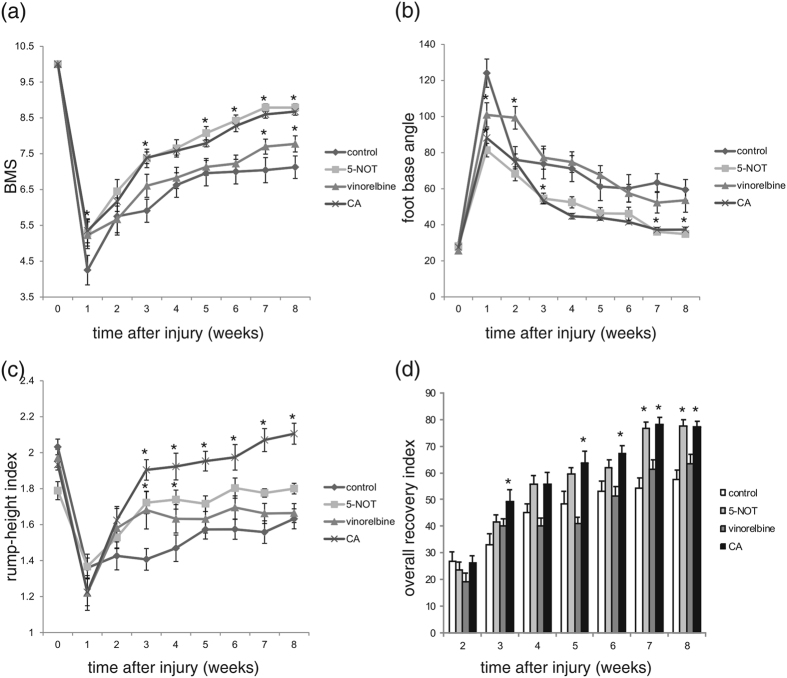
5-NOT and colominic acid enhance recovery after spinal cord injury in mice. Time course of motor recovery after spinal cord lesion. Shown are mean values ± SEM of BMS (**a**), foot-base angle (**b**), rump-height index (**c**) and overall recovery index (**d**) at different time-points after spinal cord injury and application of vehicle control, colominic acid (CA), 5-NOT or vinorelbine (n = 8 per group). Asterisks indicate differences between vehicle control, colominic acid, 5-NOT or vinorelbine treated groups after injury (*p < 0.05, two-way ANOVA with Holm-Sidak post-hoc test).

5-NOT and vinorelbine were tested in a mouse model of spinal cord injury with immediate application after injury and directly into the lesion site. Compound concentrations used were slightly higher than those with highest impact on neurite growth and neuronal migration *in vitro*^26^,^27^ and in the range of the PSA mimicking compound tegaserod^21^. Colominic acid was applied in parallel to compare the effects of PSA and the PSA mimicking compounds. BMS scoring, determination of foot-base angles and rump-height indices were performed before injury and thereafter weekly for up to eight weeks (Fig. 1). Three days after injury all mice showed flaccid hind limb paralysis demonstrating that the spinal cord lesion was complete. From the first week after injury locomotor function was improved in the colominic acid and 5-NOT treated animals compared to the mice that had received the vehicle control, whereas vinorelbine-treated animals showed improved locomotion at seven and eight weeks after injury (Fig. 1a). Foot-stepping angles as an estimate of stepping quality also improved more rapidly in mice treated with 5-NOT and colominic acid, whereas vinorelbine-treated and control animals were not different (Fig. 1b). For the rump-height index as an indicator of body weight support during ground locomotion, recovery was improved in colominic acid-treated mice at three weeks after injury and at all later time-points. 5-NOT and vinorelbine treated mice recovered better at three and four weeks after injury, but not at later time-points (Fig. 1c). We also calculated individual recovery indexes (RI) (degree of return of function, 100% indicating complete recovery) for each of the three parameters and mean of these RI per animal designated overall recovery index. At 7 and 8 weeks after injury, the overall RI of 5-NOT treated mice was significantly higher than in mice treated with vinorelbine or in control animals. The overall recovery index in colominic acid treated mice was significantly higher than the overall recovery index of control animals at all time-points after the fifth week (Fig. 1d). In conclusion, treatment with colominic acid and 5-NOT, but not with vinorelbine, improves the functional outcome compared with control treatment, although not completely restoring walking capabilities.

### Application of 5-NOT, vinorelbine and colominic acid enhances expression of neurofilament in the vicinity of the lesion site, without changing microglial/macrophage responses and astrogliosis

**Figure 2:**
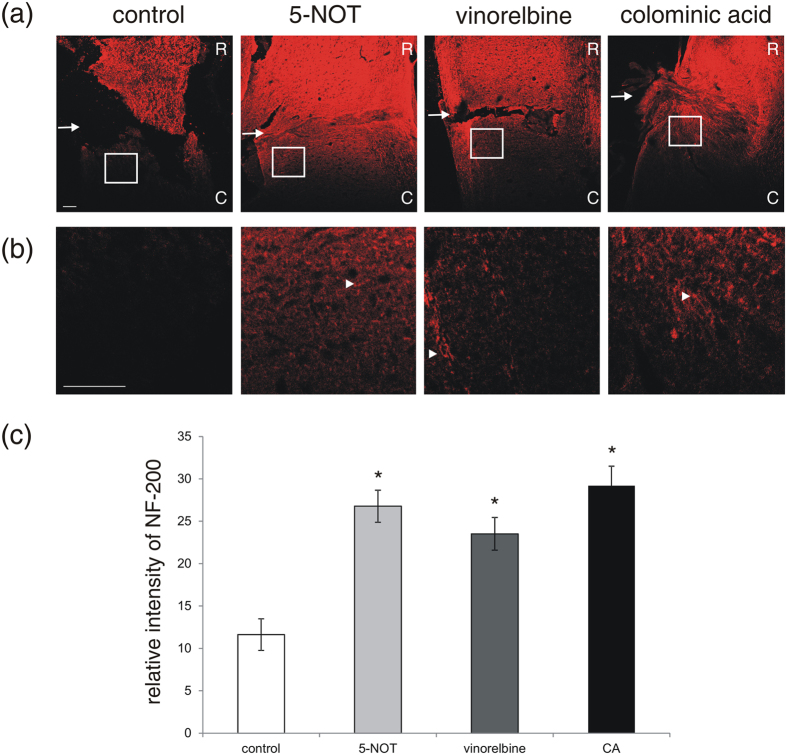
5-NOT, colominic acid and vinorelbine enhance expression of NF-200 in the vicinity of lesion site. (**a,b**) Laser scanning images of NF-200^+^ neurites projecting into the lesion site (R: rostral side; C: caudal side) in sagittal spinal cord sections. (**a**) Low magnification images. Arrows depict the lesion site. (**b**) High magnification images of the boxed areas in (**a**) are shown. Arrowheads point to NF-200 immuno-positive fibers. (**c**) NF-200 immunofluorescence intensity (mean values ± SEM, n = 4 per group) in the vicinity and within the lesion site in 5-NOT, vinorelbine and colominic acid (CA) treated mice as compared to vehicle control. (*p < 0.05, one-way ANOVA with Holm-Sidak post-hoc test). Scale bars: 100 μm.

Eight weeks after injury, neurofilament 200-positive (NF-200^+^) neurites projecting into the lesion site were seen in all groups and their numbers were not different between groups. Overall expression of NF-200 around the lesion site, especially caudally to the lesion, was significantly higher in 5-NOT, vinorelbine and colominic acid treated mice as compared to control mice (Fig. 2). Increase in neurofilament density in the vicinity of the lesion site has been accepted as a reliable indicator of axonal regrowth and verifies successful regrowth resulting from application of PSA and PSA mimetics.

Number of activated Iba1 positive microglia/macrophages as identified by their compact morphology tended to be lower in colominic acid treated mice and higher in vinorelbine treated mice. Although differences were not significant compared to control animals, there was statistically significant difference between colominic acid and vinorelbine (Supplementary Fig. 1). With regard to GFAP expression no difference in expression of GFAP at or surrounding the lesion area in all groups of mice was detectable (Supplementary Fig. 2). The size of the GFAP immunopositive glial scar was not different between groups (data not shown).

### Protection of motor neurons caudal to the injury site by 5-NOT, vinorelbine and colominic acid

**Figure 3:**
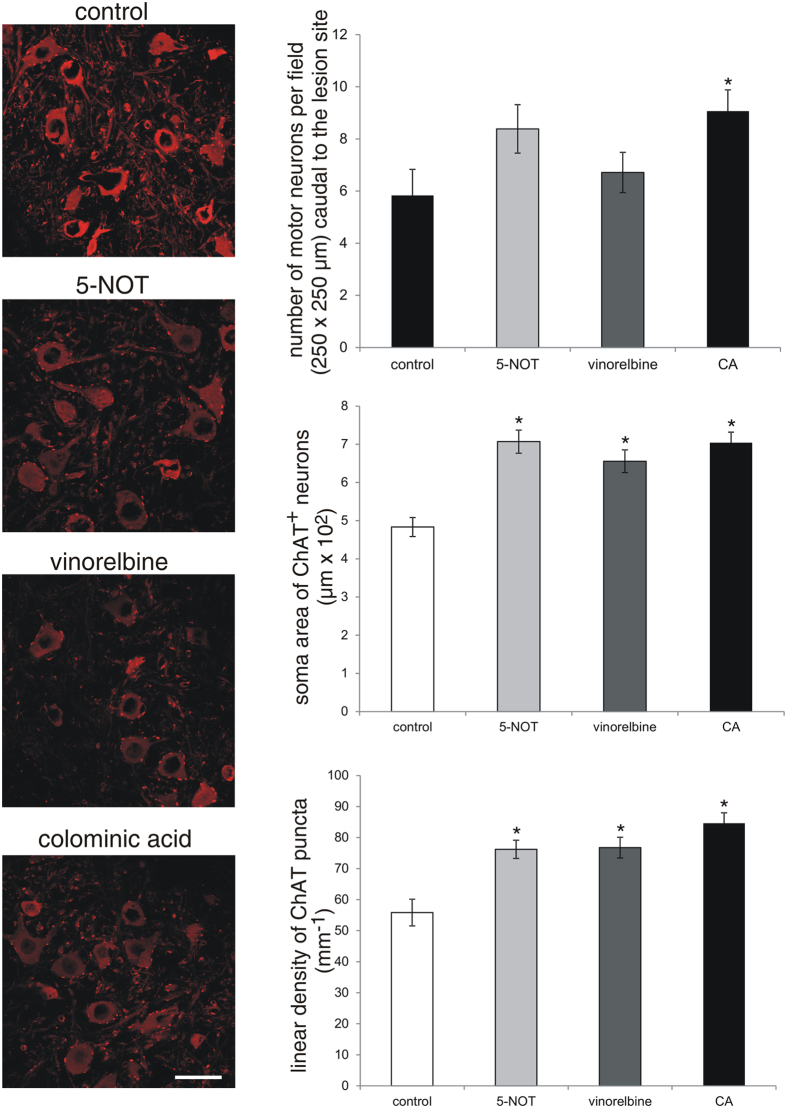
Application of 5-NOT, colominic acid and vinorelbine increases the soma area and linear density of ChAT^+^ perisomatic puncta. Confocal images (1 μm-thick optical sections) showing ChAT^+^ perisomatic puncta around motor neurons 250 μm caudal to the lesion site in sagittal spinal cord sections. Soma area of ChAT^+^ neurons, number of ChAT^+^ motor neurons and linear density of ChAT^+^ puncta (mean values ± SEM, n = 4 per group) around motor neurons in 5-NOT, vinorelbine and colominic acid (CA) treated mice as compared to vehicle control. (*p < 0.05, one-way ANOVA with Holm-Sidak post-hoc test). Scale bar: 50 μm.

**Figure 4:**
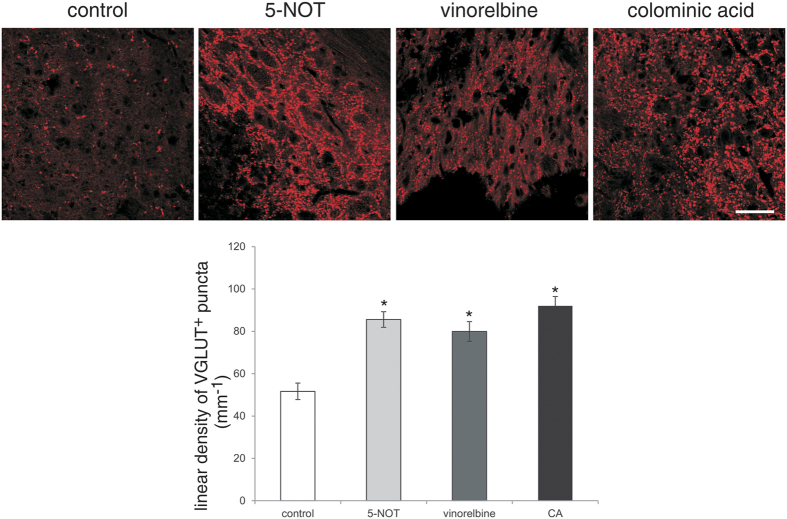
Application of 5-NOT, colominic acid and vinorelbine increases linear density of VGLUT1^+^ perisomatic puncta. Confocal images showing VGLUT^+^ perisomatic puncta around motor neurons 250 μm caudal to the lesion site in sagittal spinal cord sections. Linear density of VGLUT^+^ puncta (mean values ± SEM, n = 4 per group) around motor neurons in 5-NOT, vinorelbine and colominic acid (CA) treated mice as compared to vehicle control. (*p < 0.05, one-way ANOVA with Holm-Sidak post-hoc test). Scale bar: 50 μm.

**Figure 5:**
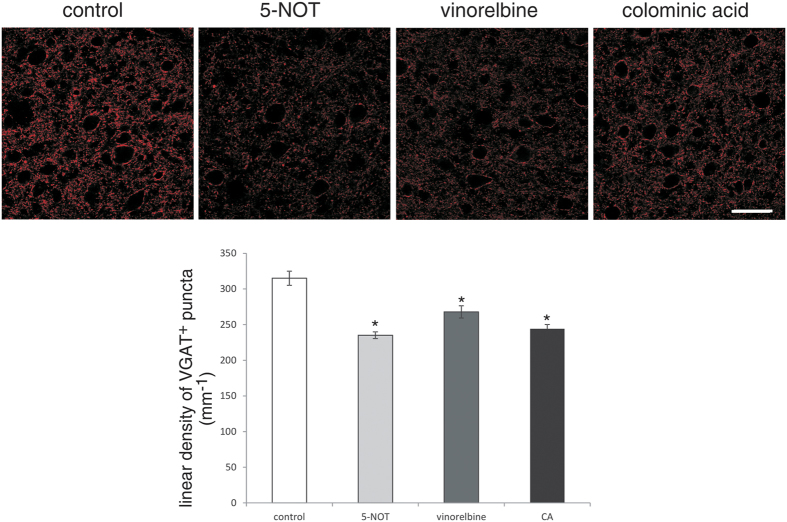
Application of 5-NOT, colominic acid and vinorelbine decreases linear density of VGAT^+^ perisomatic puncta. Confocal images of 1 μm-thick optical sections showing VGAT^+^ perisomatic puncta around motor neurons 250 μm caudal to the lesion site in sagittal spinal cord sections. Linear density of VGAT^+^ puncta (mean values ± SEM, n = 4 per group) around motor neurons in 5-NOT, vinorelbine and colominic acid (CA) treated mice as compared to vehicle control. (*p < 0.05, one-way ANOVA with Holm-Sidak post-hoc test). Scale bar: 50 μm.

Soma size of motor neurons caudal to the injury site, as estimated by the area of choline acetyltransferase-positive (ChAT^+^) cell bodies, was smaller in control animals than in 5-NOT, vinorelbine and colominic acid treated mice. Also, an increase in the linear density of perisomatic ChAT buttons was observed in 5-NOT, vinorelbine and colominic acid treated mice (Fig. 3). A considerable increase in the number of motor neurons within an area of 250 × 250 μm^2^, 250 μm caudally to the lesion site was observed in mice treated with colominic acid. Although not significant, application of 5-NOT tended to show a higher number of motor neurons than the control and vinorelbine groups (Fig. 3). As an indicator for protection of proprioceptive afferent synapses from motor neurons, the linear density of vesicular glutamate transporter1 (VGLUT1) puncta on motor neuron cell bodies was increased in 5-NOT, vinorelbine and colominic acid treated mice compared to control animals (Fig. 4). The linear density of vesicular GABA transporter-positive (VGAT^+^) puncta at motor neuron cell bodies, as indicator of GABA mediated presynaptic inhibition, was reduced in 5-NOT, vinorelbine and colominic acid treated animals when compared to control mice (Fig. 5). These results indicate that colominic acid and PSA mimetics increase synaptic and structural plasticity.

### 5-NOT enhances chatecholaminergic reinnervation caudal to the injury site

Monoaminergic innervation as measured by tyrosine hydroxylase-immunoreactive (TH^+^) axons originating from the brain stem^28^ and projecting beyond an arbitrarily selected border 250 μm caudal to the lesion site after eight weeks of injury was not different between control mice and mice treated with colominic acid, 5-NOT or vinorelbine. In addition, numbers were higher in 5-NOT treated mice compared to vinorelbine treated mice which exhibited the lowest number of TH^+^ axons beyond the lesion site (Supplementary Fig. 3). Since monoaminergic innervation of the spinal cord controls the coordinated, rhythmic movements and is important for locomotor functions^29^, a higher number of TH^+^ axons in the 5-NOT treated mice may indicate its beneficial effects on recovery.

### 5-NOT, vinorelbine and colominic acid enhance re-myelination

**Figure 6:**
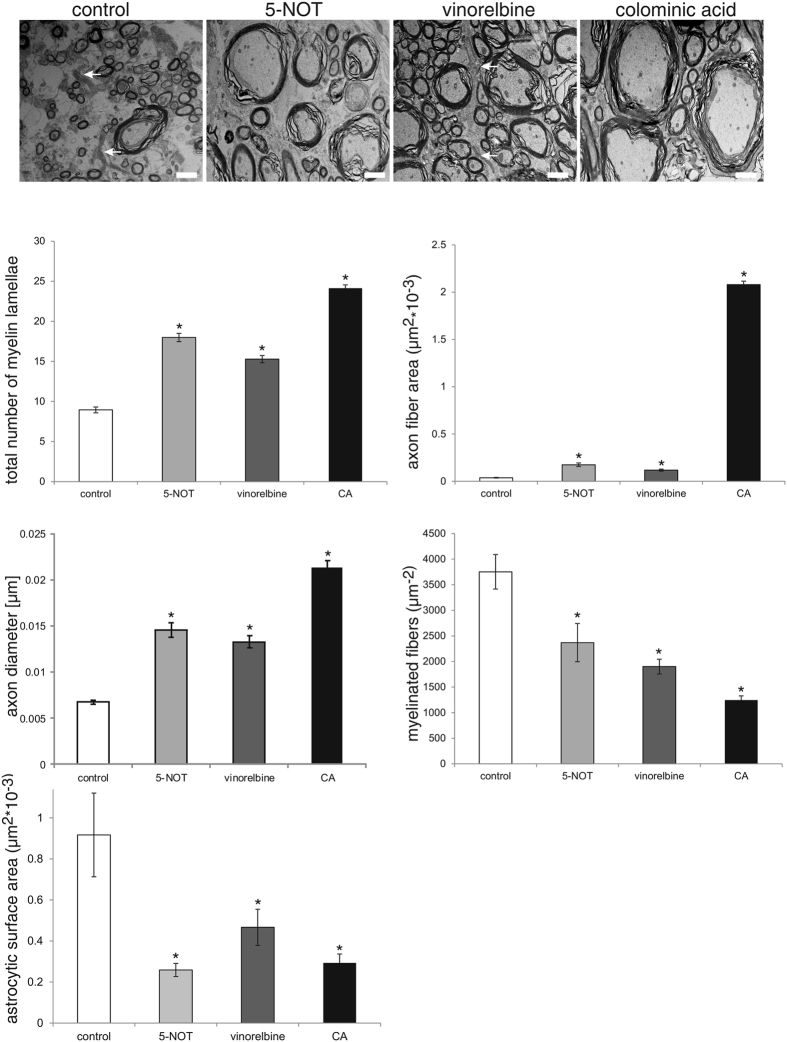
Application of 5-NOT, colominic acid and vinorelbine increases number of myelin lamellae and cross-sectional area of axons. Electron microscopic images show axons 10 μm caudal to the lesion site in transverse spinal cord sections. Number of myelin lamellae (mean values ± SEM, n = 4 per group) around axons, the cross-sectional area of axons (mean values ± SEM, n = 4 per group), axon diameters (mean values ± SEM, n = 4 per group), numbers of myelinated fibers (mean values ± SEM, n = 4 per group) and astrocytic surface area (mean values ± SEM, n = 4 per group) in 5-NOT, vinorelbine and colominic acid (CA) treated mice as compared to vehicle control. (*p < 0.05, one-way ANOVA with Holm-Sidak post-hoc test). Arrows denote astrocytic processes. Scale bar: 2 μm.

Since remyelination is presumed to promote recovery after injury, we determined the number of myelin lamellae around axon fibers and the area of axon fibers after SCI as a measure for remyelination, axonal survival and recovery after injury. Compared to myelin sheaths of control animals, myelin sheaths of 5-NOT, vinorelbine and colominic acid treated mice were thicker and even smaller caliber axons were re-myelinated in 5-NOT, vinorelbine and colominic acid treated mice (Fig. 6). There was no difference in the number of myelin sheaths around axon fibers in 5-NOT, vinorelbine and colominic acid treated mice. Additionally, cross sectional calibers of axons were larger in 5-NOT, vinorelbine and colominic acid treated mice. In particular, application of colominic acid led to a strong increase in axon fiber area which was also higher than in 5-NOT and vinorelbine treated mice. Furthermore, colominic acid application decreased the number of myelinated fibers per area not only compared to control animals but also compared to 5-NOT and vinorelbine treated mice (Fig. 6). Ultrastructural studies also revealed high numbers of thick astrocytic processes surrounding myelinated and unmyelinated fibers in the control group. Although some astrocytic processes were also seen in mice treated with 5-NOT, vinorelbine or colominic acid, the astrocytic cell surface area was reduced when compared to non-treated control mice (Fig. 6).

## Discussion

Previous studies showed that 5-NOT, a known serotonin receptor_1B/1D_ agonist, and vinorelbine, a βIII-tubulin binding cytostatic drug approved for treatment of non-small cell lung cancer and breast cancer, can mimic PSA and enhance beneficial neuronal functions *in vitro*^26^,^27^. In the present study, we compared the compounds with the polysialic acid homolog colominic acid, in promoting functional recovery after spinal cord injury in mice. We could show that after spinal cord injury, the restorative efficacy of 5-NOT was similar to the efficacy of colominic acid in adult mice with spinal cord injury, whereas vinorelbine was less effective. Topical exposure of 5-NOT to the lesion site immediately after injury enhanced locomotor recovery to a similar extent as colominic acid treatment. Although, in some of the evaluated parameters, such as rump-height index, the axon fiber area and the number of myelinated fibers, colominic acid appeared more effective than the PSA mimetics, the combined outcomes of 5-NOT treatment were similar to those of colominic acid.

Other parameters describing beneficial outcomes of the small compound application showed that 5-NOT, vinorelbine and colominic acid enhanced neurofilament immunoreactivity in the vicinity of the lesion site. Enhanced expression of neurofilament correlates with the beneficial outcome seen with application of small compounds. Spinal cord injury is often accompanied by degradation and loss of neurofilament proteins^30^,^31^,^32^. Cell based approaches using transplantation of neurally induced bone marrow derived mesenchymal stem cells^33^ or neurotrophin-3-expressing bone mesenchymal stem cells^34^ into the injured rat spinal cord have shown improved neurofilament immunoreactivity and functional outcome.

Spinal cord injury leads to severe atrophy of rubrospinal and cortical motor neurons^35^,^36^. Increase in soma areas of ChAT^+^ motor neurons in 5-NOT, vinorelbine and colominic acid treated mice as compared to control mice indicates that these compounds may prevent the axotomy induced decline in the size of motor neuronal cell bodies. The linear density of perisomatic ChAT immunopositive axon terminals was also increased in mice treated with colominic acid, 5-NOT and vinorelbine. Also, C-type synapses formed by ChAT immunopositive axon terminals on motor neurons^37^ are essential for repetitive discharges^38^ and excitability of motor neurons^39^. It is therefore reasonable to assume that treatment of mice with colominic acid or the small organic compounds is conducive for motor neuronal excitability.

Spinal cord injury not only leads to degradation of neurofilament proteins but also results in the loss of motor function control mechanisms due to deafferentation. This loss is most likely prevented, since 5-NOT, vinorelbine and colominic acid lead to improved regrowth of severed axons and an increase in perisomatic VGLUT1 expression. The vesicular glutamate transporter is responsible for glutamate uptake into synaptic vesicles, thereby protecting neurons from excitotoxicity. Similar effects on recovery and/or axon regrowth after spinal cord injury were observed with polysialyltransferase-overexpressing Schwann cells, Schwann cells engineered to express PSA-NCAM, PSA mimicking peptides or lentiviral-mediated expression of PSA^40^,^16^,^17^,^13^. Inhibitory interneurons play important roles in remodeling of nervous system functions. It is therefore interesting that the VGAT is highly expressed in the nerve endings of GABAergic and glycinergic neurons of the spinal cord^41^. Moreover, several studies have suggested that GABAergic inhibitory circuits influence plasticity. GABAergic circuits are pivotal for reshuffling of cortical motor representations^42^ and for deafferentation of visual and somatosensory cortices, allowing these brain regions to retain higher levels of plasticity^43^. Based on these findings, we propose that a decrease in VGAT expression by application of PSA mimetics after spinal cord injury may indicate increased network plasticity.

After spinal cord injury a prolonged and dispersed oligodendrocyte cell death is responsible for widespread demyelination. Prolonged demyelination leads to enhanced susceptibility of axons to degeneration and contributes to functional impairment associated with spinal cord injury^44^. Remyelination following spinal cord injury is essential for improvement in locomotor function^45^. 5-NOT, vinorelbine and colominic acid treatment increased axonal diameter and number and thickness of myelin sheaths. Increased axon caliber correlates with myelin thickness^46^,^47^ and myelination is critical for determining the caliber of dorsal root ganglion neurons^48^. Myelinating Schwann cells modulate neurofilament phosphorylation and their packing density, thereby improving axon caliber expansion in peripheral nerves^49^. It was reported that astrocytic processes and the astroglial scar are responsible for the failure to remyelinate^45^. Although we did not observe differences in the expression of GFAP between groups, ultrastructural examination of spinal cord tissue showed fewer astrocytic processes in mice treated with vinorelbine than in the control group, suggesting that astrocytes and their processes may contribute to the reduced remyelination in control animals. The observed increase in remyelination and myelin thickness in mice treated with colominic acid and PSA mimetics could also be due to their effects on oligodendrocytes. PSA enhances migration and differentiation of oligodendrocyte progenitor cells^50^ and enzymatic removal of PSA disrupts the migration of oligodendrocyte precursors and leads to their premature differentiation^51^. Interestingly, although no influence of PSA on GFAP expression levels nor CSPG immunoreactivity signals caudally to the lesion site was detected eleven weeks after injury in rats grafted with PSA overexpressing Schwann cells, improved functional recovery was observed compared to control animals eleven weeks after injury^40^. Application of the PSA mimicking compound PR-21 in mouse spinal cord injury did not influence overall CSPG immunoreactivity, but enhanced locomotor recovery in parallel with numbers of serotonergic fibers^25^ suggesting in particular that enhanced axon regrowth is responsible for PSA- and PSA mimetic-mediated improved recovery.

Neither the small compounds nor colominic acid reduced the enhanced GFAP expression indicative of astrogliosis after injury, but colominic acid and PSA mimetics favored axonal regrowth, suggesting that astrogliosis does not invariably generate a hostile territory after injury in the presence of molecules favoring axonal regrowth. Although after SCI activated microglia, which contribute to the growth inhibiting environment for neurons and the development of pain, were observed around the lesion site in all animals, the expression of Iba1 in vinorelbine treated mice was significantly higher than in colominic acid treated animals. The observed differences in vinorelbine treated groups may be due to anti-proliferative and cytotoxic activities of vinca alkaloids, such as vinorelbine, on microtubule functions leading *in vivo* to peripheral neurotoxicity^52^.

In the present study, we have used a onetime intraoperational application of small compounds and colominic acid which led to protracted effects in recovery from injury. This is noteworthy, since the small organic compounds as well as the colominic acid polymer are expected to be limited in retention time in the injected tissue. Also, their diffusion in the spinal cord tissue cannot be controlled (see Supplementary Table 1 for the available drug and pharmacological information regarding vinorelbine and 5-NOT). Nevertheless, it is conceivable that application of these compounds during the acute tissue reaction to injury beneficially modulates the initial cellular and molecular reactions, thereby generating an environment that protects the impaired tissue and allows repair and regeneration. Such effects after a single application of a polysialic acid mimicking peptide have been reported to serve in functional gain after peripheral nerve injury^20^. Although, due to presence of sialidases in the nervous system^53^,^54^ colominic acid could be quickly degraded, we propose that colominic acid can act rapidly and efficiently in inducing early plastic and molecular changes in the injured spinal cord that are conducive to functional recovery. Interestingly, regain of locomotor functions are observed more early in colominic acid and 5-NOT treated mice than in vinorelbine treated animals. Cytotoxicity and anti-proliferative effects of vinorelbine may influence and hamper this initial repair process.

Neurological diseases and, in particular, mental health disorders, become increasingly recognized as the health challenge of the 21^st^ century. Despite considerable investments, development of new therapies to treat acute and chronic nervous system disease or injury has been slow. Aiming at repurposing of the well characterized drug 5-NOT for non-nervous system indications builds on available safety and pharmacology profiles to develop a therapy exploiting it for the treatment of nervous system diseases^55^. The PSA-mimetic 5-NOT has not been FDA approved for patient treatment, but has been suggested as therapeutic agent to treat hepatitis and cancer (US patents US 20100255001 A1 and US 20140296278 A1). Re-purposing of this drug for novel applications should be feasible, although in the majority of cases, a drug is repurposed for a different indication based on its known mechanism of action. However, histone deacetylase inhibitors, which are currently used for treatment of cancer, were suggested to be re-purposed for treatment of stroke and white matter ischemic injury^56^. Furthermore, drugs to treat diabetes have been proposed to be retasked for the treatment of Alzheimer’s disease^57^.

In comparison to colominic acid, 5-NOT is similar in potency to stimulate regeneration after injury and 5-NOT is as effective as the PSA-mimicking peptides in enhancing regeneration after spinal cord injury^17^,^25^, but can be applied at lower concentrations and is not degraded by peptidases. Compared with the stimulatory effect of the PSA-mimicking compound tegaserod in a spinal cord injury paradigm, which yielded an overall recovery of mice of approximately 30%^18^, 5-NOT-treated mice recovered to a higher extent (80% recovery). Since the mode of delivery of test compounds in the two studies was different and since the Alzet pump may have mechanically influenced locomotor behavior, a comparison between the two studies is not possible. Nevertheless, we consider a higher recovery index after a single intraoperational application of 5-NOT to be encouraging. These results show that 5-NOT is a promising drug for the development of novel nervous system therapies.

The combined data support the view that application of the PSA mimetic 5-NOT creates favorable conditions for regrowing axons and re-myelination to enhance functional recovery. We would like to suggest that this compound could participate in a combinational therapeutic strategy for promoting the repair of acute nervous system injuries and neurodegenerative diseases.

## Materials and Methods

### Mice

Female C57BL/6J mice were obtained from the breeding facility of the University Hospital Hamburg-Eppendorf. Mice were kept at standard laboratory conditions with food and water supply ad libitum and with an artificial 12 h light/dark cycle. All experiments were conducted in accordance with the German and European Community laws on protection of experimental animals, and all procedures used were approved by the responsible committee of the State of Hamburg (animal permit numbers ORG 679 Morph and 98/09).

### Antibodies and reagents

Purchased reagents are indicated with their companies in brackets: monoclonal mouse antibody against neurofilament (NF-200, 1:1,000 Sigma-Aldrich, St. Louis, MO), monoclonal mouse antibody against vesicular GABA transporter (VGAT, 1:1,000; Synaptic Systems, Göttingen, Germany), polyclonal rabbit antibody against glial fibrillary acidic protein (GFAP, 1:1,000; Dako, Hamburg, Germany), polyclonal rabbit anti-ionized calcium-binding adapter molecule 1 (Iba-1; 1:1,000; Wako Chemicals, Neuss, Germany), polyclonal rabbit antibody against tyrosine hydroxylase (TH, 1:800; Chemicon, Hofheim, Germany), polyclonal rabbit antibody against vesicular glutamate transporter 1 (VGLUT1, 1:1,000; Synaptic Systems), polyclonal goat antibody against choline acetyltransferase (ChAT, 1:100; Merck Millipore, Darmstadt, Germany), secondary antibodies coupled with Cy2 or Cy3 (Jackson ImmunoResearch, Newmarket, UK).

### Application of 5-NOT and vinorelbine to injured spinal cords

Spinal cord injury was performed as described^17^ using three-month-old female C57BL/6J mice. For surgery, mice were anesthetized by intraperitoneal injections of ketamin and xylazin (100 mg Ketanest, Parke-Davis/Pfizer, Karlsruhe, Germany, and 5 mg Rompun, Bayer, Leverkusen, Germany, per kg body weight). Laminectomy was performed at the T7–T9 level with mouse laminectomy forceps (Fine Science Tools, Heidelberg, Germany). A mouse spinal cord compression device was used to elicit compression injury. Compression force (degree of closure of the forceps) and duration were controlled by an electromagnetic device: the spinal cord was maximally compressed (100%, according to the operational definition of Curtis *et al*.^58^ for 1 second by a time-controlled current flow through the electromagnetic device. PBS, colominic acid (40 μg/ml), vinorelbine (200 nM) and 5-NOT (200 nM) were applied via a very thin pulled out single barrel microfilament with an outside diameter <0.5 mm and inside diameter <0.3 mm (AM Systems) to inject the volume of 2 μl into the spinal cord 1 mm proximally and distally to the lesion site (eight animals per group). This method leads to a minimal lesion injury due to the fine geometry of the microcapillary^59^. Muscles and skin were then closed using 6–0 nylon stitches (Ethicon, Norderstedt, Germany). After the surgery, mice were kept on a heated pad (37 °C) for several hours to prevent hypothermia and thereafter singly housed in a temperature-controlled (22 °C) room with water and soft food. During the postoperative period the bladders of the animals were manually voided twice daily.

### Functional metrics of regeneration

The recovery of ground locomotion after spinal cord injury was evaluated using the Basso mouse scale (BMS)^60^. In addition, we used a more complex and objective assessment of locomotion, single-frame motion analysis as described^20^. This approach includes evaluation of beam walking [foot-base angle (FBA), rump-height index (RHI)]. The FBA is defined by a line parallel to the dorsal surface of the hind paw and the horizontal line. The angle is measured with respect to the posterior aspect at the beginning of the stance phase. In intact mice, this phase is well defined and the angle is approximately 20°. After spinal cord injury and severe loss of locomotor abilities, the mice drag behind their hindlimbs with dorsal paw surfaces facing the beam surface. The angle is increased to >150°. The second parameter, the RHI, was estimated from the recordings used for measurements of the foot-stepping angle. The parameter is defined as height of the rump, i.e., the vertical distance from the dorsal aspect of the animal’s tail base to the beam, normalized to the thickness of the beam measured along the same vertical line. Assessment was performed before and at every week after the injury. Values for the left and right extremities were averaged. Recovery indices (RI) were used as a measure of functional recovery at the individual animal level. The RI is calculated (percentage) as follows:

RI = [(X7 + n − X7)/(X0 − X7)] × 100, where X0, X7, and X7 + n are values before operation, 7 days after injury, and a time-point n days after the spinal cord injury, respectively. This measure estimates gain of function (X7 + n − X7) as a fraction of the functional loss (X0 − X7) induced by the injury. Overall RI were calculated, on an individual animal basis, as means of RI for the three parameters: BMS, foot-stepping angle and rump-height index.

### Histology

Mice were anesthetized by intraperitoneal injection of 16% sodium pentobarbital solution (Narcoren, Merial, Hallbergmoos, Germany, 5 µl/g body weight) and were transcardially perfused with 4% formaldehyde in 0.1 M sodium cacodylate buffer, pH 7.3. The spinal cord was removed two hours after fixing, post-fixed overnight at 4 °C and then immersed in 15% sucrose solution in 0.1 M cacodylate buffer, pH 7.3, for 1 day at 4 °C. Afterwards the tissue was frozen for 2 min in 2-methyl-butane (isopentane, Carl Roth, Karlsruhe, Germany) pre-cooled to −80 °C. For sectioning, the spinal cord segment was attached to a cryostat specimen holder using TissueTek (Sakura Finetek Europe, Zoeterwoude, The Netherlands). Serial transverse or parasagittal sections of 25 μm thickness were cut on a cryostat (Leica CM3050, Leica Instruments, Nußloch, Germany) and picked up on Super Frost Plus glass slides (Roth, Karlsruhe, Germany). Sampling of sections was always done in a standard sequence so that four sections 250 μm apart were present on each slide. Immunohistochemistry was performed as described earlier^61^.

### Immunohistochemistry

Water bath antigen de-masking was performed in 10 mM sodium citrate solution, pH 9.0, for 30 minutes at 80 °C for all antigens. Nonspecific binding was blocked using 5% normal serum from the species in which the secondary antibody was produced, dissolved in PBS and supplemented with 0.2% Triton X-100, 0.02% sodium azide for 1 hour at room temperature (RT). Incubation with the primary antibody (anti-NF-200, anti-GFAP, anti-Iba1, anti-VGAT, anti-ChAT, anti-VGLUT1 or anti-tyrosine hydroxylase), diluted in PBS containing 0.5% lambda-carrageenan (Sigma-Aldrich) and 0.02% sodium azide, was carried out for 3 days at 4 °C. After washing in PBS (3 × 15 minutes at RT), the appropriate secondary antibody, diluted 1:200 in PBS-carrageenan solution, was applied for 2 hours at RT. After a subsequent wash in PBS, the sections were mounted in antiquenching medium (Fluoromount/DAPI; Carl Roth, Germany) and stored in the dark at 4 °C. Photographic documentation was made on an LSM 510 confocal microscope (Carl Zeiss, Oberkochen, Germany) or an Axiophot two microscope equipped with a digital camera AxioCam HRc and AxioVision software (Zeiss). The images were processed using ImageJ software (NIH, Bethesda, MD).

To evaluate the immunofluorescence intensity of NF-200, GFAP and Iba1, microscopic images were captured (six longitudinal sections per animal and four ROIs per section) from the rostral and caudal regions in the immediate vicinity of the lesion site by confocal microscopy (LSM 150, Carl Zeiss) and converted into grey scale after consistent threshold adjustment using ImageJ software (NIH, Bethesda, MD) to measure their intensity profiles. For evaluation of perisomatic terminals, longitudinal spinal cord sections immunostained for ChAT, VGLUT and VGAT were examined by confocal microscopy (LSM 150, Carl Zeiss) and optical sectioning was used for recording of 1-μm-stacks with an oil immersion objective (63 × 2). Six stacks per region were evaluated. One image per cell was chosen on the basis of the largest cross-sectional cell body area. The cell soma perimeter was measured and the number of perisomatic terminals was counted using ImageJ software (NIH, Bethesda, MD). Linear density of the perisomatic puncta was calculated as number of counted perisomatic terminals per unit length. The Axiophot microscope (Carl Zeiss) equipped with a motorized stage and Neurolucida software-controlled computer system (MicroBrightField, Magdeburg, Germany) was used to count the number of TH-positive axons 250 μm caudally to the lesion site.

### Electron microscopy

Spinal cords were dissected from perfused animals. The tissue samples were post-fixed in 1% osmium tetroxide (Polysciences Europe, Eppelheim, Germany) in 0.1 M sodium cacodylate buffer, pH 7.3, for 1 hour at RT, dehydrated in ascending ethanol solutions and embedded in resin according to standard protocols. For ultrastructural analysis, 50 nm-thick cross-sections 10 μm caudally to the lesion site were cut in a sequential manner, from three 5 μm-equidistant points. Sections were subjected to transmission electron microscopy and numbers of myelinated axons, myelinated axonal areas and numbers of myelin sheaths were counted. The indicated parameters were assessed in the area of the dorsal lemniscal system, in particular the regions of fasciculus gracilis and cuneatus. Hundred sections from three different animals per group were evaluated. Counting was performed by two independent researchers, blind to the treatments.

### Statistical analysis

All numerical data are presented as group mean values with standard error of the mean (SEM) values. Parametric or nonparametric tests (one-way or two-way ANOVA with subsequent Holm-Sidak post-hoc tests) were used for comparison as appropriate. Analyses were performed using the Sigmastat v 3.5 software. The threshold value for acceptance of differences between groups was 5%.

## Additional Information

**How to cite this article**: Saini, V. *et al*. The polysialic acid mimetics 5-nonyloxytryptamine and vinorelbine facilitate nervous system repair. *Sci. Rep.* **6**, 26927; doi: 10.1038/srep26927 (2016).

## Supplementary Material

Supplementary Information

## References

[b1] Finne J., Finne U., Deagostini-Bazin H. & Goridis C. Occurrence of alpha 2–8 linked polysialosyl units in a neural cell adhesion molecule. Biochem. Biophys. Res. Commun. 112, 482–487 (1983). 6847662 10.1016/0006-291x(83)91490-0

[b2] Schnaar R. L., Gerardy-Schahn R. & Hildebrandt H. Sialic acids in the brain: gangliosides and polysialic acid in nervous system development, stability, disease, and regeneration. Physiol. Rev. 94, 461–518 (2014). 24692354 10.1152/physrev.00033.2013 PMC4044301

[b3] Roche P. H. . Expression of cell adhesion molecules in normal nerves, chronic axonal neuropathies and Schwann cell tumors. J. Neurol. Sci. 151, 127–133 (1997). 9349666 10.1016/s0022-510x(97)00110-x

[b4] Durbec P. & Cremer H. Revisiting the function of PSA-NCAM in the nervous system. Mol. Neurobiol. 24, 53–64 (2001). 11831554 10.1385/MN:24:1-3:053

[b5] Angata K. & Fukuda M. Roles of polysialic acid in migration and differentiation of neural stem cells. Methods Enzymol. 479, 25–36 (2010). 20816158 10.1016/S0076-6879(10)79002-9

[b6] Storms S. D. & Rutishauser U. A role for polysialic acid in neural cell adhesion molecule heterophilic binding to proteoglycans. J. Biol. Chem. 273, 27124–27129 (1998). 9765230 10.1074/jbc.273.42.27124

[b7] Muller D. . Brain-derived neurotrophic factor restores long-term potentiation in polysialic acid-neural cell adhesion molecule-deficient hippocampus. Proc. Natl. Acad. Sci. USA 97, 4315–4320 (2000). 10760298 10.1073/pnas.070022697 PMC18239

[b8] Vaithianathan T. . Neural cell adhesion molecule-associated polysialic acid potentiates alpha-amino-3-hydroxy-5-methylisoxazole-4-propionic acid receptor currents. J. Biol. Chem. 279, 47975–47984 (2004). 15317811 10.1074/jbc.M407138200

[b9] Hammond M. S. . Neural cell adhesion molecule-associated polysialic acid inhibits NR2B-containing N-methyl-D-aspartate receptors and prevents glutamate-induced cell death. J. Biol. Chem. 281, 34859–34869 (2006). 16987814 10.1074/jbc.M602568200

[b10] Mishra B. . Functional role of the interaction between polysialic acid and extracellular histone H1. J. Neurosci. 30, 12400–12413 (2010). 20844135 10.1523/JNEUROSCI.6407-09.2010 PMC6633434

[b11] Theis T. . Functional role of the interaction between polysialic acid and myristoylated alanine-rich C kinase substrate at the plasma membrane. J. Biol. Chem. 288, 6726–6742 (2013). 23329829 10.1074/jbc.M112.444034 PMC3585110

[b12] El Maarouf A., Petridis A. K. & Rutishauser U. Use of polysialic acid in repair of the central nervous system. Proc. Natl. Acad. Sci. USA 103, 16989–16994 (2006). 17075041 10.1073/pnas.0608036103 PMC1636566

[b13] Zhang Y. . Lentiviral-mediated expression of polysialic acid in spinal cord and conditioning lesion promote regeneration of sensory axons into spinal cord. Mol. Ther. 15, 1796–1804 (2007). 17551503 10.1038/sj.mt.6300220

[b14] Zhang Y. . Induced expression of polysialic acid in the spinal cord promotes regeneration of sensory axons. Mol. Cell. Neurosci. 35, 109–119 (2007). 17363265 10.1016/j.mcn.2007.02.011

[b15] Zhang Y., Zhang X., Yeh J., Richardson P. & Bo X. Engineered expression of polysialic acid enhances Purkinje cell axonal regeneration in L1/GAP-43 double transgenic mice. Eur. J. Neurosci. 25, 351–361 (2007). 17284175 10.1111/j.1460-9568.2007.05311.x

[b16] Papastefanaki F. . Grafts of Schwann cells engineered to express PSA-NCAM promote functional recovery after spinal cord injury. Brain 130, 2159–2174 (2007). 17626035 10.1093/brain/awm155

[b17] Mehanna A. . Polysialic acid glycomimetic promotes functional recovery and plasticity after spinal cord injury in mice. Mol. Ther. 18, 34–43 (2010). 19826404 10.1038/mt.2009.235 PMC2839208

[b18] Pan H. C., Shen Y. Q., Loers G., Jakovcevski I. & Schachner M. Tegaserod, a small compound mimetic of polysialic acid, promotes functional recovery after spinal cord injury in mice. Neuroscience 277, 356–366 (2014). 25014876 10.1016/j.neuroscience.2014.06.069

[b19] Jungnickel J. . Polysialyltransferase-overexpression in Schwann cells mediates different effects during peripheral nerve regeneration. Glycobiology 22, 107–115 (2012). 21840969 10.1093/glycob/cwr113

[b20] Mehanna A. . Polysialic acid glycomimetics promote myelination and functional recovery after peripheral nerve injury in mice. Brain 132, 1449–1462 (2009). 19454531 10.1093/brain/awp128

[b21] Bushman J. . Tegaserod mimics the neurostimulatory glycan polysialic acid and promotes nervous system repair. Neuropharmacology 79, 456–466 (2014). 24067923 10.1016/j.neuropharm.2013.09.014 PMC4618794

[b22] Kim H. S. . PSA-NCAM(+) neural precursor cells from human embryonic stem cells promote neural tissue integrity and behavioral performance in a rat stroke model. Stem Cell Rev. 10, 761–771 (2014). 24974101 10.1007/s12015-014-9535-y

[b23] Franceschini I. . Migrating and myelinating potential of neural precursors engineered to overexpress PSA-NCAM. Mol. Cell. Neurosci. 27, 151–162 (2004). 15485771 10.1016/j.mcn.2004.05.006

[b24] Falconer R. A., Errington R. J., Shnyder S. D., Smith P. J. & Patterson L. H. Polysialyltransferase: a new target in metastatic cancer. Curr. Cancer Drug Targets 12, 925–939 (2012). 22463390 10.2174/156800912803251225

[b25] Marino P., Norreel J. C., Schachner M., Rougon G. & Amoureux M. C. A polysialic acid mimetic peptide promotes functional recovery in a mouse model of spinal cord injury. Exp. Neurol. 219, 163–174 (2009). 19445935 10.1016/j.expneurol.2009.05.009

[b26] Loers G. . Nonyloxytryptamine mimics polysialic acid and modulates neuronal and glial functions in cell culture. J. Neurochem. 128, 88–100 (2014). 23957498 10.1111/jnc.12408

[b27] Loers G. . Vinorelbine and epirubicin share common features with polysialic acid and modulate neuronal and glial functions. J. Neurochem. 136, 48–62 (2016). 26443186 10.1111/jnc.13383 PMC4904230

[b28] Loers G., Cui Y. F., Neumaier I., Schachner M. & Skerra A. A Fab fragment directed against the neural cell adhesion molecule L1 enhances functional recovery after injury of the adult mouse spinal cord. Biochem. J. 460, 437–46 (2014). 24673421 10.1042/BJ20131677

[b29] Cui Y. F. . Embryonic stem cell derived L1 overexpressing neural aggregates enhance recovery after spinal cord injury in mice. PLoS One 6, e17126 (2011). 21445247 10.1371/journal.pone.0017126 PMC3060805

[b30] Yoshimura N., Erdman S. L., Snider M. W. & de Groat W. C. Effects of spinal cord injury on neurofilament immunoreactivity and capasaicin sensitivity in rat dorsal root ganglion neurons innervating the urinary bladder. Neuroscience 83, 633–643 (1998). 9460769 10.1016/s0306-4522(97)00376-x

[b31] Banik N. L., Matzelle D. C., Gantt-Wilford G., Osborne A. & Hogan E. L. Increased calpain content and progressive degradation of neurofilament protein in spinal cord injury. Brain Res. 752, 301–306 (1997). 9106471 10.1016/s0006-8993(96)01488-6

[b32] Banik N. L., Hogan E. L., Powers J. M. & Whetstine L. J. Degradation of cytoskeletal proteins in experimental spinal cord injury. Neurochem. Res. 7, 1465–1475 (1982). 7170062 10.1007/BF00965089

[b33] Yazdani S. O. . A comparison between neurally induced bone marrow derived mesenchymal stem cells and olfactory ensheathing glial cells to repair spinal cord injuries in rat. Tissue Cell 44, 205–213 (2012). 22551686 10.1016/j.tice.2012.03.003

[b34] Wang L. J., Zhang R. P. & Li J. D. Transplantation of neurotrophin-3-expressing bone mesenchymal stem cells improves recovery in a rat model of spinal cord injury. Acta Neurochir. (Wien) 7, 1409–1418 (2014). 24744011 10.1007/s00701-014-2089-6

[b35] Bregman B. S., Broude E., McAtee M. & Kelley M. S. Transplants and neurotrophic factors prevent atrophy of mature CNS neurons after spinal cord injury. Exp. Neurol. 149, 13–27 (1998). 9454611 10.1006/exnr.1997.6669

[b36] Hains B. C., Black J. A. & Waxman S. G. Primary cortical motor neurons undergo apoptosis after axotomizing spinal cord injury. J. Comp. Neurol. 462, 328–341 (2003). 12794736 10.1002/cne.10733

[b37] Davidoff M. S. & Irintchev A. P. Acetylcholinestrase activity and type C synapses in the hypoglossal, facial and spinal-cord motor nuclei of rats. An electron-microscope study. Histochemistry 84, 515–524 (1986). 3721918 10.1007/BF00482985

[b38] Chevallier S., Nagy F. & Cabelguen J. M. Cholinergic control of excitability of spinal motoneurons in the salamander. J. Physiol. 570, 525–540 (2006). 16308350 10.1113/jphysiol.2005.098970 PMC1479874

[b39] Miles G. B., Hartley R., Todd A. J. & Brownstone R. M. Spinal cholinergic interneurons regulate the excitability of motoneurons during locomotion. Proc. Natl. Acad. Sci. USA 104, 2448–2453 (2007). 17287343 10.1073/pnas.0611134104 PMC1794344

[b40] Gosh M. . Extensive cell migration, axon regeneration, and improved function with polysialic acid-modified Schwann cells after spinal cord injury. Glia 60, 979–992 (2012). 22460918 10.1002/glia.22330 PMC4387847

[b41] Chaudhry F. A. . The vesicular GABA transporter, VGAT, localizes to synaptic vesicles in sets of glycinergic as well as GABAergic neurons. J. Neurosci. 18, 9733–9750 (1998). 9822734 10.1523/JNEUROSCI.18-23-09733.1998 PMC6793280

[b42] Jackobs K. M. & Donoghue J. P. Reshaping the cortical motor map by unmasking latent intracortical connections. Science 251, 944–947 (1991). 2000496 10.1126/science.2000496

[b43] Ding Y., Kastin A. J. & Pan W. Neural plasticity after spinal cord injury. Curr. Pharm. Des. 11, 1441–1450 (2005). 15853674 10.2174/1381612053507855 PMC3562709

[b44] Bhatt A., Fan L. W. & Pang Y. Strategies for myelin regeneration: lessons learned from development. Neural Regen. Res . 9, 1347–1350 (2014). 25221590 10.4103/1673-5374.137586 PMC4160864

[b45] Keirstead H. S. . Human embryonic stem cell-derived oligodendrocyte progenitor cell transplants remyelinate and restore locomotion after spinal cord injury. J. Neurosci. 25, 4694–4705 (2005). 15888645 10.1523/JNEUROSCI.0311-05.2005 PMC6724772

[b46] Aguayo A. J., Epps J., Charron L. & Bray G. M. Multipotentiality of Schwann cells in cross-anastomosed and grafted myelinated and unmyelinated nerves: quantitative microscopy and autoradiography. Brain Res. 104, 1–20 (1976). 1247896 10.1016/0006-8993(76)90643-0

[b47] Weinberg H. J. & Spencer P. S. Studies on the control of myelinogenesis. II. Evidence for neuronal regulation of myelin production. Brain Res. 113, 363–378 (1976). 953741 10.1016/0006-8993(76)90947-1

[b48] Windebank A. J., Wood P., Bunge R. P. & Dyck P. J. Myelination determines the caliber of dorsal root ganglion neurons in culture. J. Neurosci. 5, 1563–1569 (1985). 4009246 10.1523/JNEUROSCI.05-06-01563.1985 PMC6565257

[b49] Nixon R. A., Paskevich P. A., Sihag R. K. & Thayer C. Y. Phosphorylation on carboxyl terminus domains of neurofilament proteins in retinal ganglion cell neurons *in vivo*: influences on regional neurofilament accumulation, interneurofilament spacing, and axon caliber. J. Cell Biol. 126, 1031–1046 (1994). 7519617 10.1083/jcb.126.4.1031 PMC2120120

[b50] Rutishauser U. Polysialic acid in the plasticity of the developing and adult vertebrate nervous system. Nat. Rev. Neurosci. 9, 26–35 (2008). 18059411 10.1038/nrn2285

[b51] Decker L., Avellana-Adalid V., Nait-Oumesmar B., Durbec P. & Baron-Van Evercooren A. Oligodendrocyte precursor migration and differentiation: combined effects of PSA residues, growth factors, and substrates. Mol. Cell Neurosci. 16, 422–439 (2000). 11085879 10.1006/mcne.2000.0885

[b52] Moudi M., Go R., Yien C. Y. S. & Nazre M. Vinca alkaloids. Int. J. Prev. Med. 4, 1231–1235 (2013). 24404355 PMC3883245

[b53] Takahashi K. . Sialidase NEU4 hydrolyzes polysialic acids of neural cell adhesion molecules and negatively regulates neurite formation by hippocampal neurons. J. Biol. Chem. 287, 14816–14826 (2012). 22393058 10.1074/jbc.M111.324186 PMC3340223

[b54] Sumida M. . Rapid trimming of cell surface polysialic acid (PolySia) by exovesicular sialidase triggers release of preexisting surface neurotrophin. J. Biol. Chem. 290, 13202–13214 (2015). 25750127 10.1074/jbc.M115.638759 PMC4505574

[b55] Chong C. R. & Sullivan D. J. New uses for old drugs. Nature 448, 645–646 (2007). 17687303 10.1038/448645a

[b56] Baltan S., Morrison R. S. & Murphy S. P. Novel protective effects of histone deacetylase inhibition on stroke and white matter ischemic injury. Neurotherapeutics 10, 798–807 (2013). 23881453 10.1007/s13311-013-0201-x PMC3805855

[b57] Yarchoan M. & Arnold S. E. Repurposing diabetes drugs for brain insulin resistance in Alzheimer disease. Diabetes 63, 2253–2261 (2014). 24931035 10.2337/db14-0287 PMC4066335

[b58] Curtis R., Green D., Lindsay R. M. & Wilkin G. P. Up-regulation of GAP-43 and growth of axons in rat spinal cord after compression injury. J. Neurocytol. 22, 51–64 (1993). 8426193 10.1007/BF01183975

[b59] Lutz D. . Myelin basic protein cleaves cell adhesion molecule L1 and improves regeneration after injury. Mol. Neurobiol. 2015 Jun 17. [Epub ahead of print]. 10.1007/s12035-015-9277-0 26081148

[b60] Basso D. M. . Basso Mouse Scale for locomotion detects differences in recovery after spinal cord injury in five common mouse strains. J. Neurotrauma. 23, 635–659 (2006). 16689667 10.1089/neu.2006.23.635

[b61] Irintchev A., Rollenhagen A., Troncoso E., Kiss J. Z. & Schachner M. Structural and functional aberrations in the cerebral cortex of tenascin-C deficient mice. Cereb. Cortex. 15, 950–962 (2005). 15537675 10.1093/cercor/bhh195

