# Supplementary material for: The polysialic acid mimetics 5-nonyloxytryptamine and vinorelbine facilitate nervous system repair
Source: Sci Rep. 2016 Jun 21;6:26927. doi: 10.1038/srep26927 (PMC4914991; doi:10.1038/srep26927)
Supplement: Supplementary Information [file srep26927-s1.doc]

**The polysialic acid mimetics 5-nonyloxytryptamine and vinorelbine facilitate nervous system repair**

Vedangana Saini^1,2#^, David Lutz^2#^, Hardeep Kataria^2^, Gurcharan Kaur^1*^, Melitta Schachner^3,4*^, Gabriele Loers^2^

^1^ Department of Biotechnology, Guru Nanak Dev University, GT Road, 143005 Amritsar, India

^2^ Center for Molecular Neurobiology, University Hospital Hamburg-Eppendorf, D-20246 Hamburg, Germany

^3^ Keck Center for Collaborative Neurosciences, Rutgers University, Piscataway, NJ 08854, USA

**^4^** Center for Neuroscience, Shantou University Medical College, Shantou, Guangdong 515041, People’s Republic of China

^#^ equally contributing authors

**Supplementary Figures**


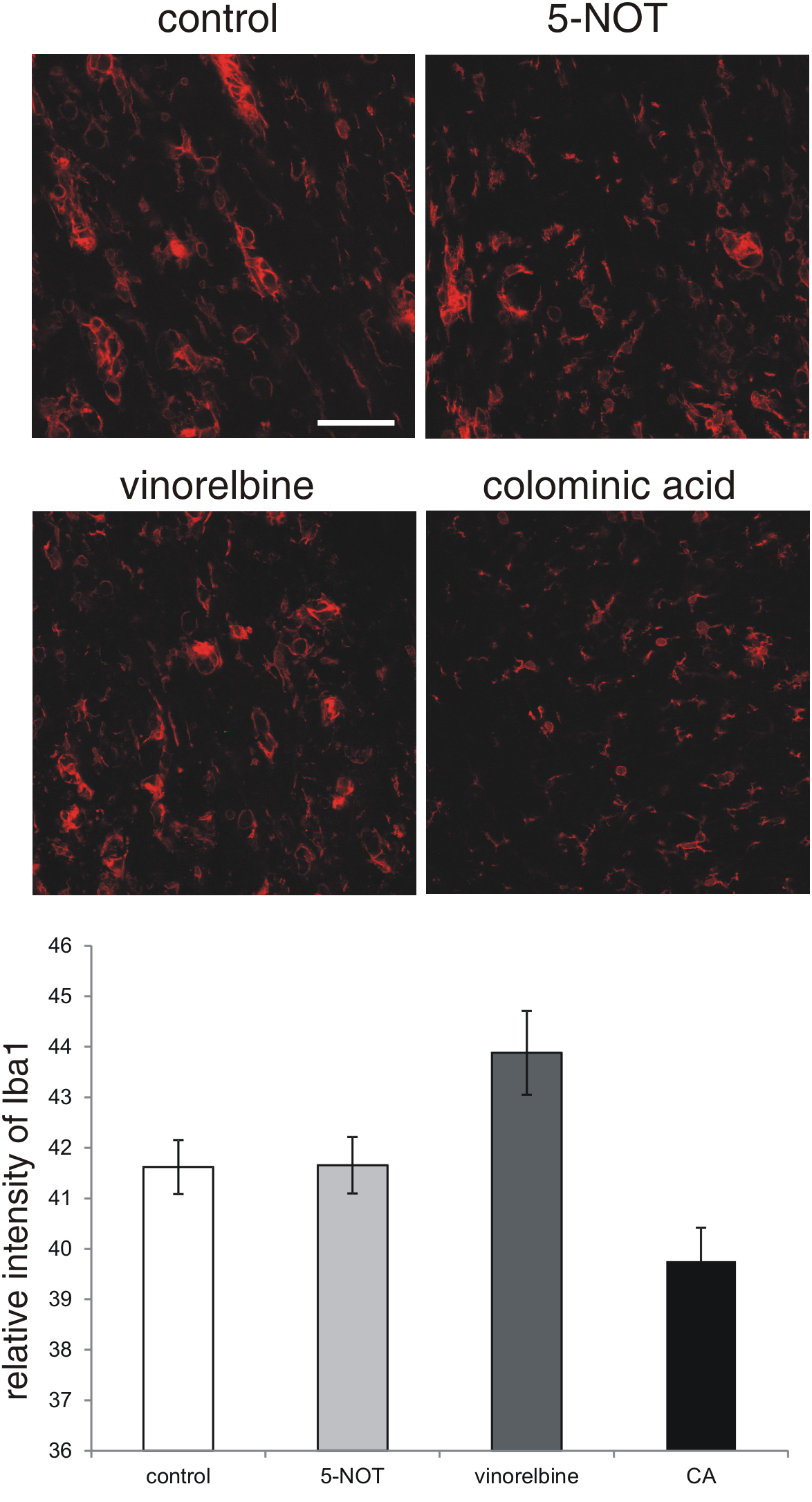


**Supplementary Figure S1. Colominic acid, 5-NOT and vinorelbine do not affect the expression of Iba1 at and near the lesion site.** Laser scanning images of Iba1^+^ cells in the vicinity of the lesion site in sagittal spinal cord sections. Mean Iba1 immunofluorescence intensity values ± SEM (n = 4 per group) in 5-NOT, vinorelbine and colominic acid (CA) treated mice as compared to vehicle control. (*p < 0.05, one-way ANOVA with Holm-Sidak post-hoc test). Scale bar: 50 µm.


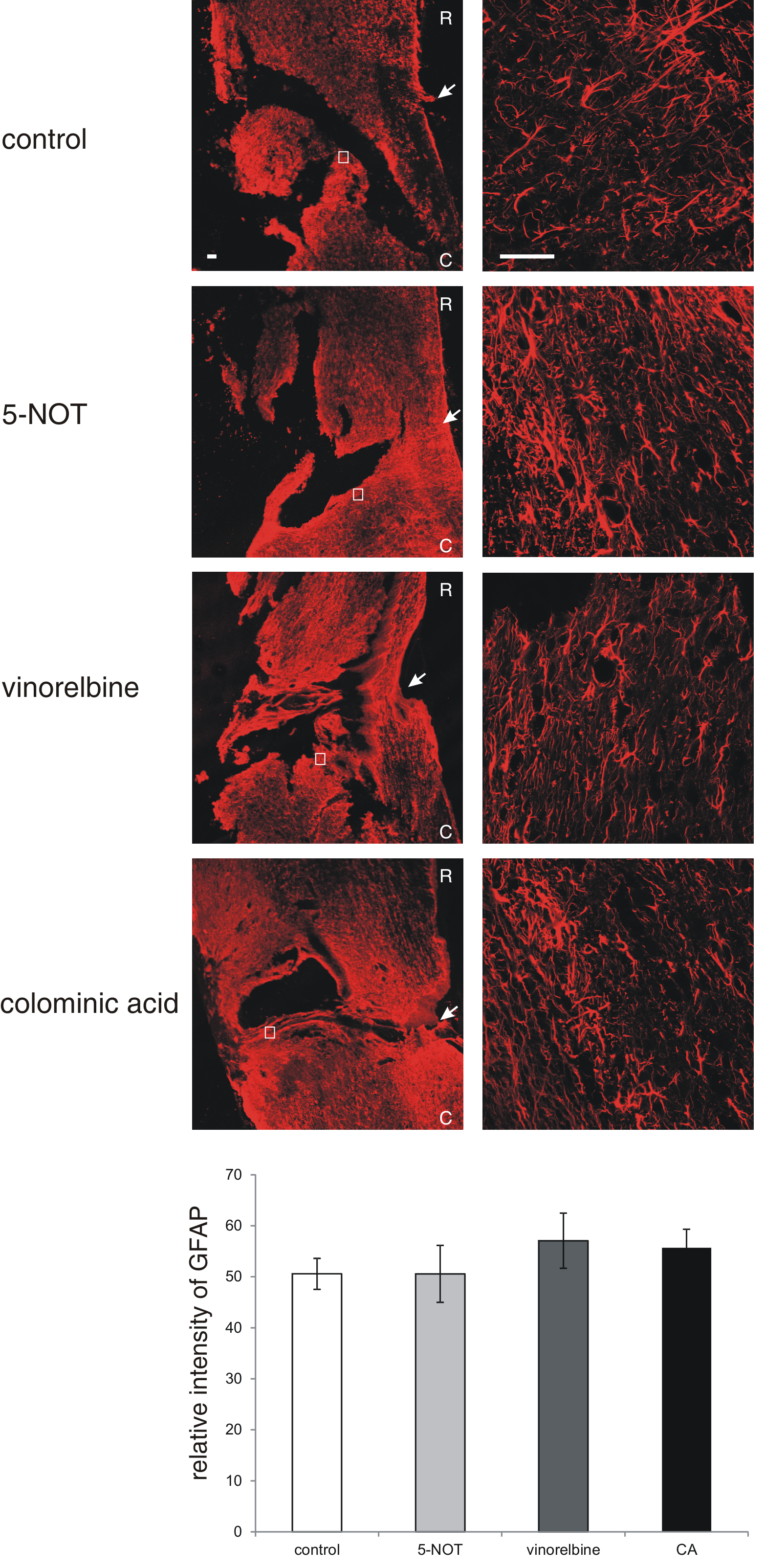


**Supplementary Figure S2. Colominic acid, 5-NOT and vinorelbine do not affect the expression of GFAP at and near the lesion site.** Laser scanning images of GFAP^+^ cells at and in the vicinity of the lesion site in sagittal spinal cord sections. On the right: high magnification images of the boxed areas in the left images are shown. Arrows depict the lesion site; R: rostral to the lesion site, C: caudal to the lesion site. Mean GFAP immunofluorescence intensity values ± SEM (n = 4 per group) in 5-NOT, vinorelbine and colominic acid treated mice as compared to vehicle control. (*p < 0.05, one-way ANOVA with Holm-Sidak post-hoc test). Scale bars: 50 µm.


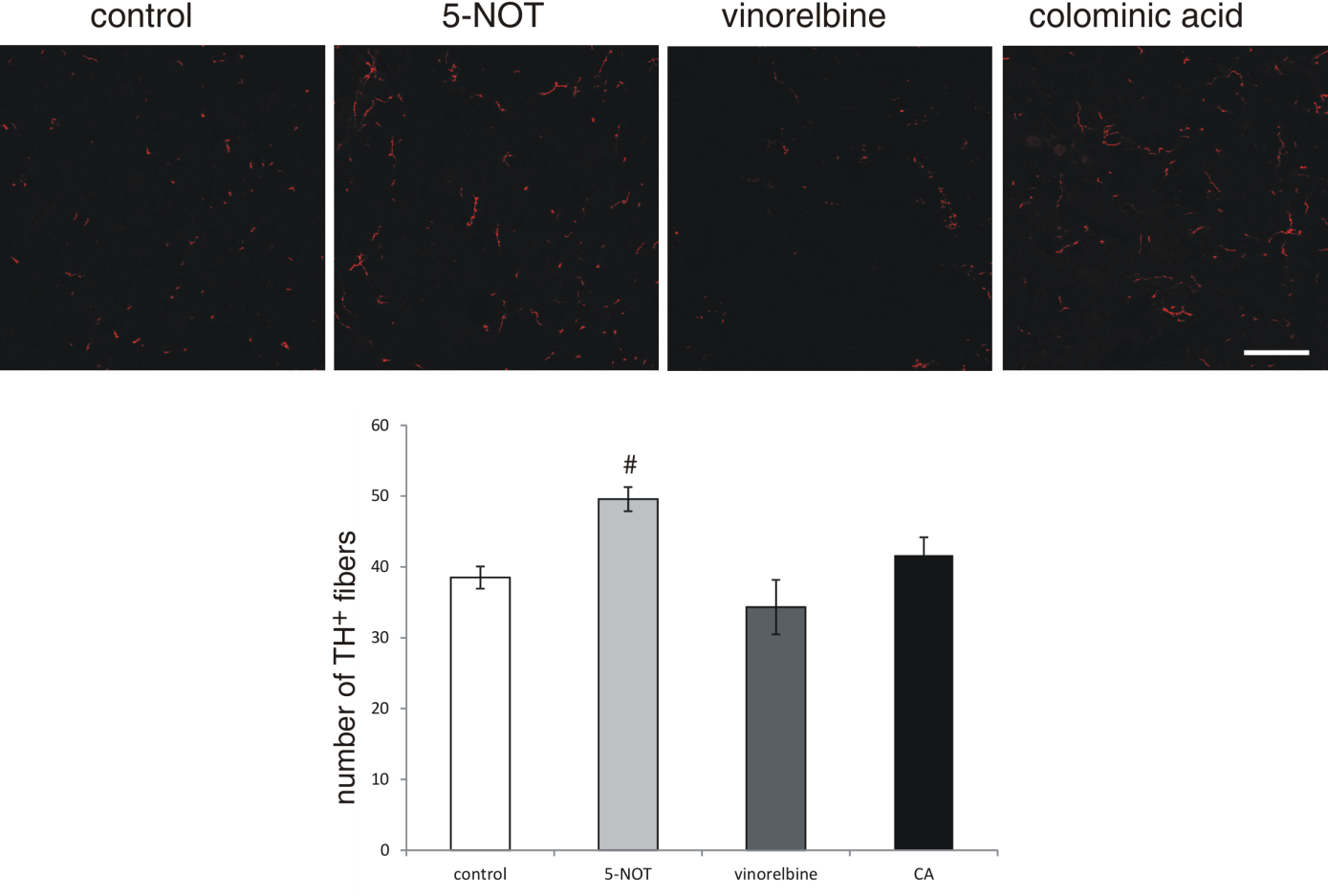


**Supplementary Figure S3. Application of 5-NOT increases the number of TH^+^ axons caudal to the lesion site.** Laser scanning images of TH^+^ axons in the spinal cord 250 µm caudal to the lesion site. Numbers (mean values ± SEM, n = 4 per group) of TH^+^ axons in 5-NOT, vinorelbine and colominic acid (CA) treated mice as compared to vehicle control. (#p < 0.05 difference between 5-NOT and vinorelbine, one-way ANOVA with Holm-Sidak post-hoc test). Scale bar: 50 µm.

**Supplementary Table**

Supplementary Table 1: Drug and pharmacological information of vinorelbine and 5-nonyloxytryptamine oxalate.

| **Drug** | **Pharmacology** | **Dosage** | **Pharmacokinetics** | **Reference** |
| --- | --- | --- | --- | --- |
| Vinorelbine  (Navelbine base) | interferes with microtubule assembly | 30 mg/m^2^ intravenous  80 mg/m^2^  oral | - triphasic concentration decay in plasma - half-life: 27.7 to 43.6 h - mean plasma clearance: 0.97 to 1.26 L/h/kg - steady-state volume of distribution 25.4 to 40.1 L/kg - peak blood concentration 133.4 ± 42.3 ng/mL^-1^ at 1.4 ± 0.7 h - absolute bioavailability 43 ± 14%   excretion 3.0 ± 2.4% | FDA  Marty et al., 2001 |
| 5-Nonyloxy-tryptamine oxalate | selective  5-HT_1B/1Dβ_ agonist | unknown | - K_i_ at 5-HT_1B/1Dβ_: 1-16 nM with 300 fold selectivity over 5-HT_1A_. - 5-NOT impairs reovirus infection and disassembly kinetics. - Stimulates binding of [^35^S]GTPϒS to G proteins in guinea pig substantia nigra by 54% at 100 µM concentration (EC_50_: 62 nM). It increased binding by 200 % in hippocampus. | Glennon et al., 1994  Mainou et al., 2015  Waeber and Moskowitz, 1997 |

**Supplementary references**

http://www.fda.gov/ohrms/dockets/ac/04/briefing/4021B1_10_Vinorelbine%20label.pdf

Glennon, R.A., Hong, S.S., Dukat, M., Teitler, M., & Davis, K. (1994) 5-(Nonyloxy)tryptamine: a novel high-affinity 5-HT1D beta serotonin receptor agonist. *J. Med. Chem*. **37**, 2828-2830.

Marty, M., Fumoleau, P., Adenis, A., Rousseau, Y., Merrouche, Y., Robinet, G., Senac, I. & Puozzo, C. (2001) Oral vinorelbine pharmacokinetics and absolute bioavailability study in patients with solid tumors. *Ann. Oncol*. **12**, 1643-1649.

Mainou, B.A., Ashbrook, A.W., Smith, E.C., Dorset, D.C., Denison, M.R. & Dermody, T.S. (2015) Serotonin receptor agonist 5-Nonyloxytryptamine alters the kinetics of reovirus cell entry. *J. Virol*. **89**, 8701-8712.

Waeber, C. & Moskowitz, M.A. (1997) 5-Hydroxytryptamine 1A and 5-hydroxytryptamine 1B receptors stimulate [35S]guanosine-5'-O-(3-thio)triphosphate binding to rodent brain sections as visualized by in vitro autoradiography. *Mol. Pharmacol*. **52**, 623-631.
